# Comparative transcriptome profiles of large and small bodied large-scale loaches cultivated in paddy fields

**DOI:** 10.1038/s41598-021-84519-9

**Published:** 2021-03-02

**Authors:** Liulan Zhao, Kuo He, Qing Xiao, Qiao Liu, Wei Luo, Jie Luo, Hongmei Fu, Jiayao Li, Xugan Wu, Jun Du, Quan Gong, Xun Wang, Song Yang

**Affiliations:** 1 College of Animal Science and Technology, Sichuan Agricultural University, Chengdu, 611130 China; 2 Engineering Research Center of Aquaculture, Shanghai Ocean University, Shanghai, 200090 China; 3 Fisheries Institute, Sichuan Academy of Agricultural Science, Chengdu, 611731 China

**Keywords:** Molecular biology, Transcriptomics

## Abstract

Fish culture in paddy fields is a traditional aquaculture mode, which has a long history in East Asia. Large-scale loach (*Paramisgurnus dabryanus*) fast growth is suitable for paddy fields aquaculture in China. The objective of this study was to identify differential expression genes (DEGs) in the brain, liver and muscle tissues between large (LG, top 5% of maximum total length) and small (SG, top 5% of minimum total length) groups using RNA-seq. In total, 150 fish were collected each week and 450 fish were collected at twelfth week from three paddy fields for all the experimental. Histological observation found that the muscle fibre diameter of LG loaches was greater than that of SG loaches. Transcriptome results revealed that the high expression genes (HEGs) in LG loaches (fold change ≥ 2, *p* < 0.05) were mainly concentrated in metabolic pathways, such as “Thyroid hormone signalling pathway”, “Citrate cycle (TCA cycle)”, “Carbon metabolism”, “Fatty acid metabolism”, and “Cholesterol metabolism”, and the HEGs in SG loaches were enriched in the pathways related to environmental information processing such as “Cell adhesion molecules (CAMs)”, “ECM− receptor interaction” and “Rap1 signalling pathway”; cellular processes such as “Tight junction”, “Focal adhesion”, “Phagosome” and “Adherens junction”. Furthermore, IGFs gene family may play an important role in loach growth for their different expression pattern between the two groups. These findings can enhance our understanding about the molecular mechanism of different growth and development levels of loaches in paddy fields.

## Introduction

Fish culture in paddy fields is an ancient and widely practiced farming model in Southeast Asia for its great advantages over traditional fish farming, such as resource conservation, environmental friendliness and economic benefits^1,2^. In most southern China areas, there are sufficient water in the paddy fields from April to October, and the water temperature during this season is suitable for the rapid growth of fish^3^. The appropriately modified paddy fields are suitable for the cultivation of some omnivorous fish such as loach and carp with large popularity. The large-scale loach (*Paramisgurnus dabryanus*) is one of the fastest growing fish species that has been widely cultivated in Southeast Asia^4^. The large-scale loach is a omnivorous species living in mild water environment, distributed in the middle and lower reaches of the Yangtze River system^5^. It is known among consumers for its richness in vitamins, minerals, essential amino acids and other trace elements meanwhile low in cholesterol^6^. The large-scale loach had been widely raised cross China as one of the most important species in paddy field aquaculture^7^. However, compared with intensive farming, the fish raised in paddy field farming has some disadvantages in terms of poor body uniformity, which will affect the market of farmed fish.

Body size is a prominent and important feature of fish in aquaculture, which is driven by muscle differentiation and growth, and it is also one of the most important factors affecting production^8^. The growth and development rate of fish varies from species to individual, and is greatly affected by environmental factors such as temperature and diet^9^. As we all know, progeny often inherit the epigenetic traits of their parents including body size and growth rate. In addition to being affected by the environmental factors, these genetic traits will also be regulated by gene function^10^. Several candidate genes that could be critical for fish growth and development have been identified from previous studies, such as neuropeptide Y (NPY) and Insulin-like growth factors (IGFs), etc.^11,12^. As the neural body center, the brain plays an important role in regulating various life activities. It can secrete a variety of growth factors (such as GH) to promote the growth of the tissues^13^, or regulate the secretion of growth factors from other tissues (such as liver)^14^. The interaction of these hormones is critical to the regulation of muscle growth, which directly determine the body size of the fish.

In this study, RNA-Seq was used to identify differentially expressed genes of three tissues (brain, liver, and muscle) between large (LG, top 5% of maximum total length) and small (SG, top 5% of minimum total length) large-scale loaches cultivated in paddy fields with the same genetic background. The purpose of this research is to improve our understanding the biological mechanism behind the loach growth as well as providing basis for future breeding at a molecular level.

## Results

### Morphological characteristics and histological observation

**Figure 1:**
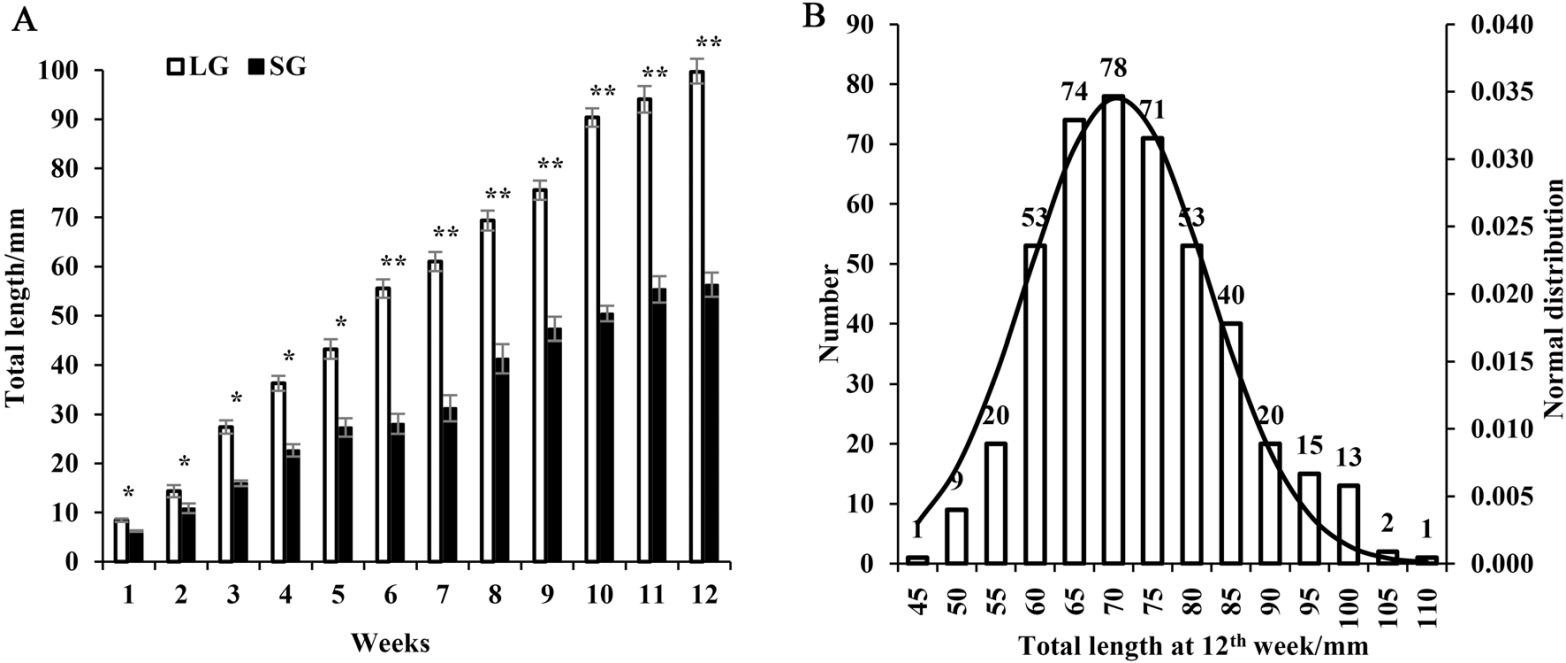
The weekly morphological characteristics (total length) of large-scale loach from large group (LG) and small group (SG). (**A**) The weekly total length of LG and SG loach (*n* = 150 fish individuals). (**B**) The normal distribution of loach total length at 12th week (*n* = 450 fish individuals). Vertical bars represent mean ± SD; * show the significant difference in total length between LG and SG; *p < 0.05, **p < 0.01.

Weekly morphological characteristics (total length) of LG fish were significantly higher than those of SG fish (*p* < 0.05), and the gap between two groups was getting more enlarge during the 12 weeks (Fig. 1A). Specifically, the total length of LG fish reached twice that of SG fish in the twelfth week (*p* < 0.05). The normal distribution of total length of large-scale loaches at week 12 was shown in Fig. 1B, and the total length was mainly concentrated from 60 to 80 cm.

**Figure 2:**
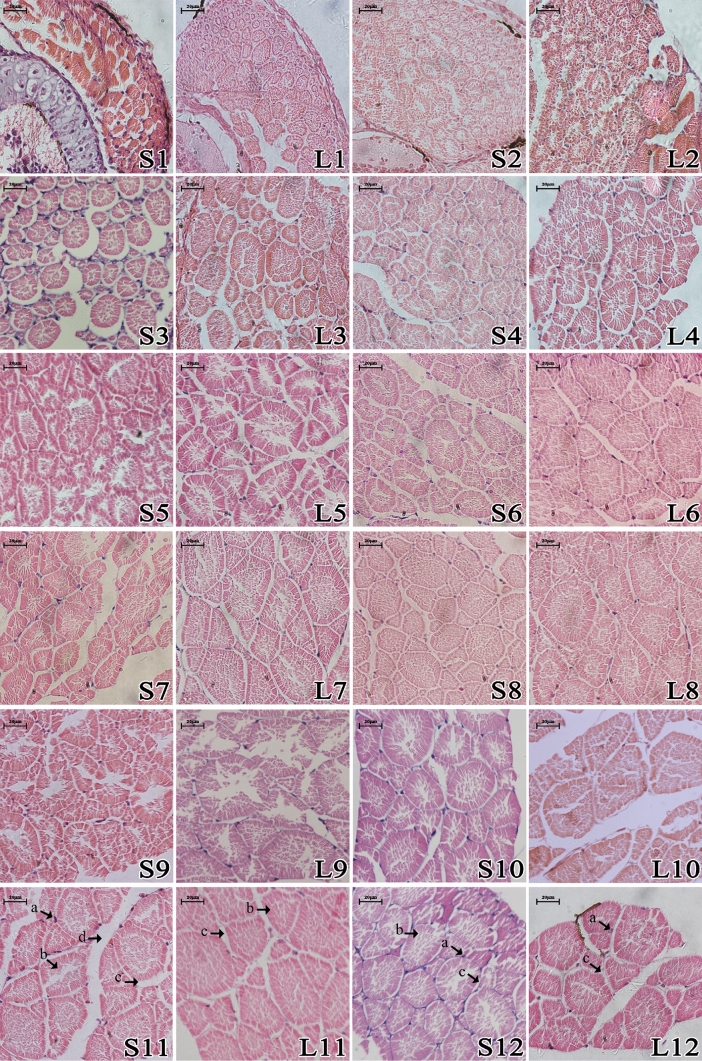
The weekly comparison of muscle histology between LG and SG loach. *Note* *L* large group, *S* small group; the number represents weeks. (a) Cell nucleus; (b) muscle fiber; (c) endomysium; (d) perimysium.

**Figure 3:**
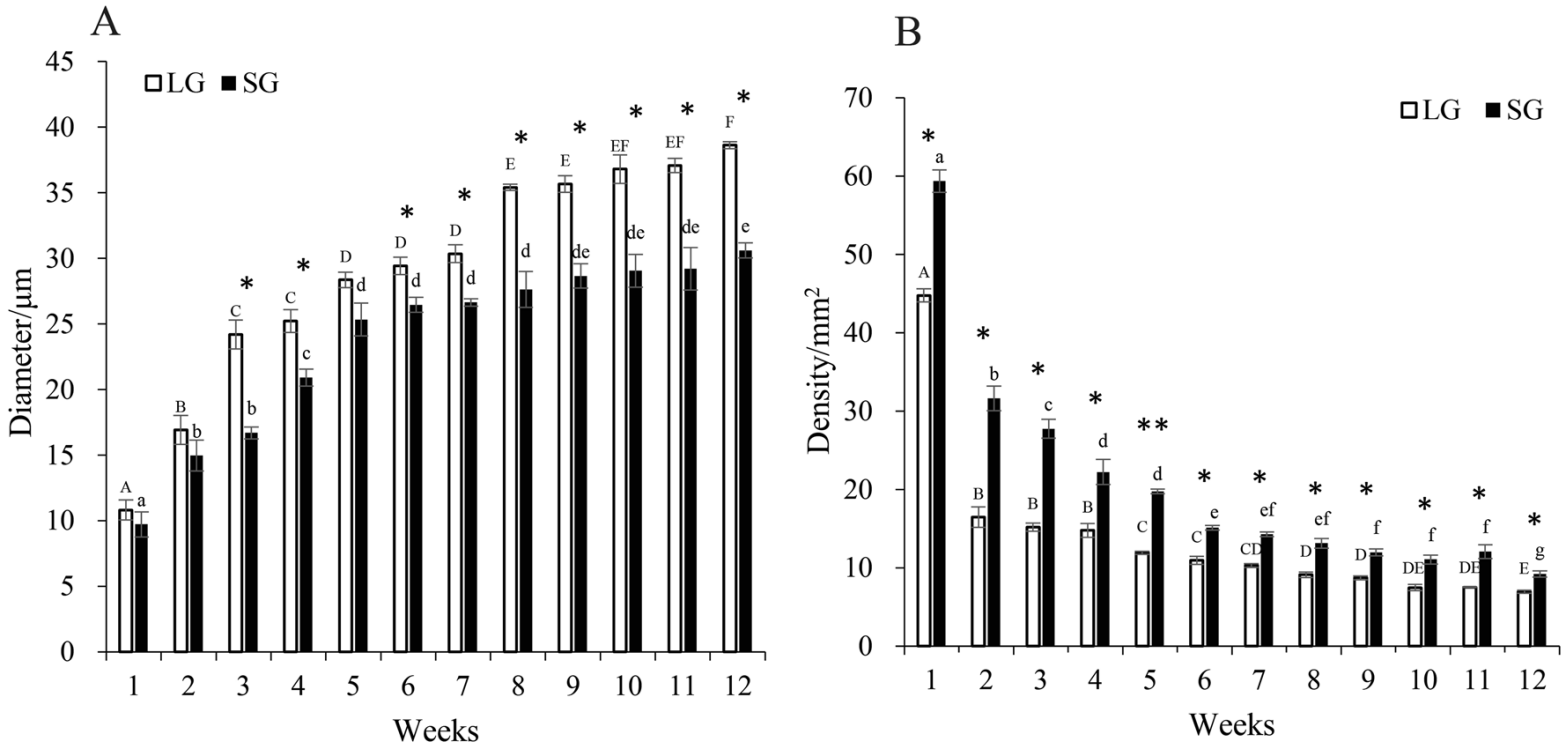
The weekly comparison of muscle fiber diameter (**A**) and density (**B**) between LG and SG loach. *Note* The different lowercase letters above the bar represent the comparison within the SG loach and the different uppercase letters represent the comparison within the LG loach. Vertical bars represent mean ± SD (*n* = 6 fish individuals); * show the significant difference in total length between LG and SG; *p < 0.05, **p < 0.01.

The comparisons of muscle histology between the LG and SG fish were presented in Fig. 2, in which we could find that the muscle fibers diameter and density were different between the two groups. Then, the muscle fibre diameter and density of two groups were measured and the statistical results were demonstrated in Fig. 3. The muscle fibers diameter was greater in the LG fish, while the density was lower in the LG (Fig. 3).

### RNA-seq data and annotation of unigenes

To identify the differentially expressed genes associated with differential body size in large-scale loach, a total of 18 cDNA libraries were constructed from LG and SG and generated about 123.63 Gb sequencing data in total. After trimming and quality control, we obtained an average of 45.79 M clean reads from the 18 cDNA libraries, and all clean Q30 base rates were over 94% (Table S1). After assembly and remove redundancy, there were 28,620 (22.52%) unigenes were over 1000 bp in length among 127,062 unigenes (mean length: 820 bp, N50: 1546 bp, percent GC: 40.90%). More detailed information of the filtered reads was presented in Table S2. Finally, 55,933 unigenes (44.02%) were functionally aligned by BLASTx against databases totally, including GO, KEGG, NCBI-nr, and UniProt.

### GO and KEGG enrichment analysis of the DEGs

**Figure 4:**
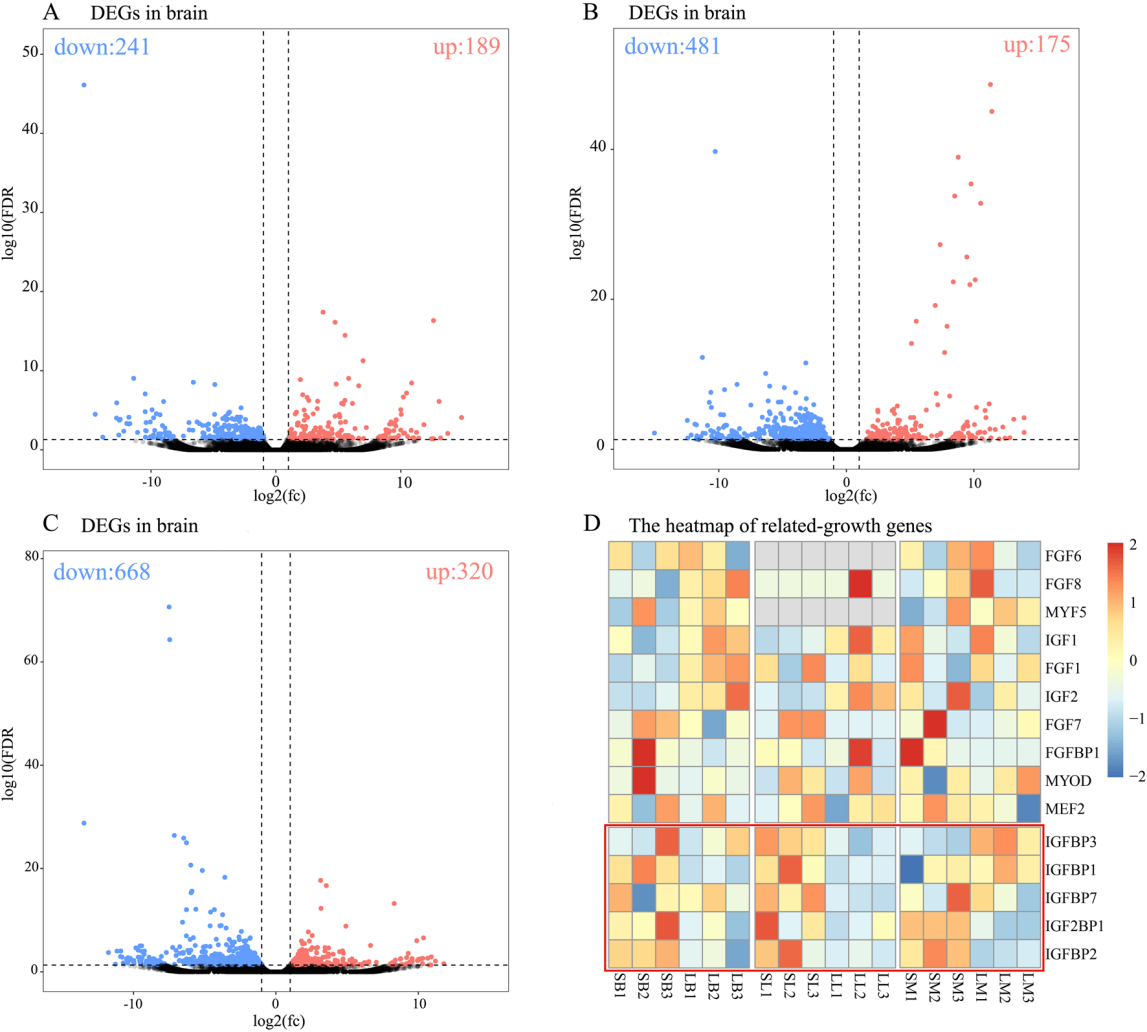
The high expressed genes (HEGs) of LG or SG loach. (**A**–**C**) the volcano map of HEGs in the brain, liver and muscle, respectively. (**D**) the heatmap of related-growth genes between LG and SG loach. LB, LL, LM: the brain, liver, and muscle of large group, SB, SL, SM: the brain, liver, and muscle of small group. *FDR* false discovery rate; *fc* fold change.

After DEGs analysis, 189, 175 and 320 high expression genes (HEGs) were identified in the LG fish brain, liver and muscle, and 241, 481 and 668 HEGs were identified in the SG fish brain, liver and muscle, respectively (Fig. 4A–C). Excluded the redundancy DEGs in two or three tissues, a total of 1861 DEGs were identified among the three samples (Supplementary excel). For the next GO and KEGG analysis, the DEGs were divided into two parts, which were composed of 684 HEGs in LG fish and 1631 HEGs in SG fish. According to Go Term and previous studies, we finally selected growth-related genes for heatmap analysis, which showed the IGFs gene family may play an important role in loach growth as their expression levels were different between the two groups (Fig. 4D). These growth-related genes were fibroblast growth factor (FGF1, 6, 7 and 8), fibroblast growth factor binding protein 1 (FGFBP1), myogenic factor 5 (MYF5), myocyte enhancer factor 2 (MEF2), myogenic differentiation (MYOD), insulin like growth factor (IGF1 and 2), insulin like growth factor binding protein (IGFBP1, 2, 3 and 7, IGF2BP1).

**Figure 5:**
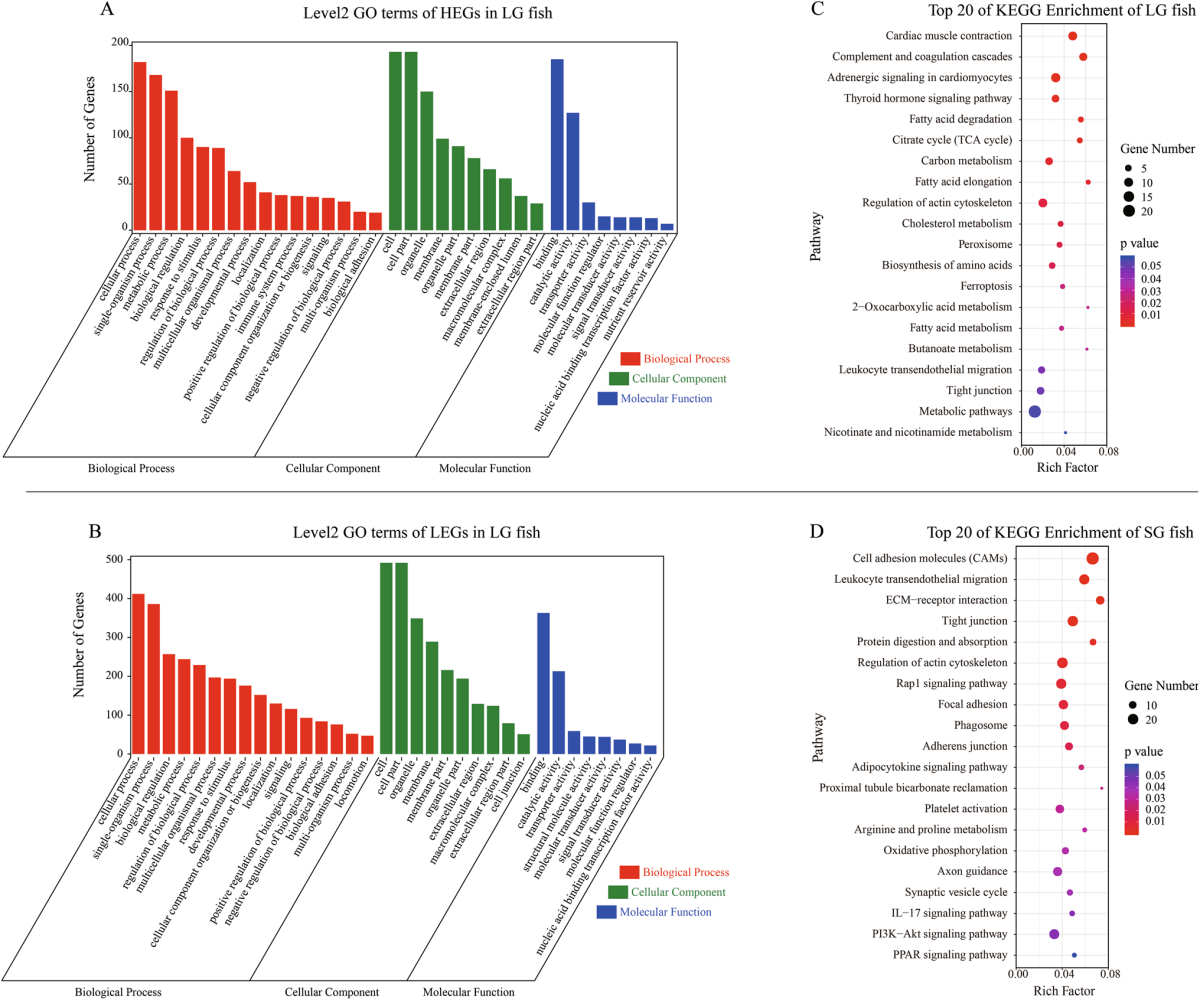
The GO annotation and KEGG pathways enrichment with HEGs of LG or SG loach. *Note* The HEGs in both groups include all the HEGs in the three tissues (brain, liver, and muscle).

GO enrichment analysis was performed to investigate the putative roles of these DEGs, which were classified into biological process (BPs), cellular component (CCs) and molecular function (MFs) categories (Fig. 5A,C). For the BPs, the major categories were cellular processes, single-organism process, metabolic process, regulation of biological processes, and cellular responses to stimulus. The major categories of the CCs were cell, cell parts, membrane parts, organelle parts and extracellular parts. The DEGs involved in binding and catalytic activity were also most represented among the MFs.

In order to identify the functional biochemical pathways of predicted proteins encoded by DEGs, KEGG pathway enrichment analysis was performed for both the HEGs in two groups (three tissues together). The top 20 of KEGG pathway enrichment analysis of two groups HEGs were showed in Fig. 5B,D, respectively. KEGG pathway enrichment analysis of HEGs in LG fish (*p* < 0.05) showed a significant enrichment of metabolic pathways, such as “Thyroid hormone signalling pathway”, “Citrate cycle (TCA cycle)”, “Carbon metabolism”, “Fatty acid metabolism”, and “Cholesterol metabolism”. The HEGs in SG fish were enriched in the pathways related to environmental information processing such as “Cell adhesion molecules (CAMs)”, “ECM–receptor interaction” and “Rap1 signalling pathway”; cellular processes such as “Tight junction”, “Focal adhesion”, “Phagosome” and “Adherens junction”.

### Validation of RNA-Seq analysis

To confirm the RNA-seq data, four genes of each tissue were selected for qRT-PCR validation. These genes included IGFBP1 (insulin-like growth factor binding protein-1), ID1 (inhibitor of DNA binding 1), MDKa (midkine a), and PPA1b (pyrophosphatase 1b). To adjust for variations in starting template, gene expression was be normalized against β-actin for each tissue, then target genes mRNA were quantified using the 2^−ΔΔCT^ method, and the level of significance was determined by one-way analysis of variance (ANOVA) with SPSS Statistics 22.0. While the relative expression levels were not perfectly consistent, the qRT-PCR can provide additional basis for the RNA-seq results (Fig. S1).

## Discussion

Intra-specific differences were first proposed by Darwin, who argued that individual differences and overproduction within a species or variety were the basis of natural selection^15^. The individual growth varies a lot in different species. The coefficient of individual growth variation in most mammals is 7%-10%, while in most fish is approximate 20%-35%^16^. Fish body size differences are mainly formed by differences in trunk skeletal muscle growth, which depends on the proliferation and hypertrophy of muscle fibers (muscle cells)^17^. During the growth process, mammalian muscle growth mainly depends on the increase of muscle fibers diameter, but less on the increase of muscle fibers quantity^18,19^. Unlike mammals and birds, fish muscle fibers maintain the ability to proliferate and hypertrophy for life^20^. For example, in larval sea bass (*Dicentrarchus labrax*), white muscle growth is mainly through differentiation to form new muscle fibers, while in larval guppy (*Poecilia reticulata*), white muscle growth is mainly achieved by increasing the size of muscle fibers^21,22^. Considering the development in other fish species, we hypothesize that loach muscles grow predominantly by means of increasing the diameter of muscle fibers in this study (Figs. 2 and 3).

The results of transcriptome sequencing revealed that the metabolism of LG loaches was more vigorous compared with that of SG loaches. The HEGs of the large loach in this study were significantly enriched in “Thyroid hormone signalling pathway”, “Citrate cycle (TCA cycle)”, “Carbon metabolism” and “Fatty acid metabolism”, and “Cholesterol metabolism”, which are all important regulators of growth, development and metabolism^23–25^. It was also found in rainbow trout that the expression of lipid-metabolism-related genes was up-regulated in fast growth fish compared with slow growth fish, which was consistent with our findings^26^. In addition, the genes related to the growth were mainly involved in energy metabolism, carbohydrate and lipid metabolism, and cytoskeletal composition, which the similar expression pattern were also observed in the study of salmonid liver and muscle gene expression^27–29^. Generally, insulin-like growth factors (IGFs) were generally thought to be up-regulated in tissues of rapidly growing individuals, such as Nile tilapia (*Oreochromis Niloticus*)^30^, channel catfish (*Ictalurus punctatus*)^31^, and Arctic charr (*Salvelinus alpinus*)^32^. It was found in this study that the gene IGF1 and IGF2 were different in two groups, but the difference was not significant (FDR > 0.05), which was similar to previous studies^11,33,34^. However, *IGFBP1* (insulin-like growth factor binding protein-1) of brain and liver and *IGFBP7* of liver expressed in SG group were both higher than that in LG loaches in this study (FDR < 0.05; Fig. 4D). In a recent study of rainbow trout, it was also found that small fish had higher expression of *IGFBP1* in the liver than in large fish^26^. Previous studies demonstrated that *IGFBP1* could inhibit *IGF* binding to cell surface receptors and thereby inhibit IGF-mediated mitogenic and cell metabolic actions^35^. Likewise, overexpression of *IGFBP1a/b* in zebrafish would retard embryonic development and growth^36,37^. Therefore, in addition to having stronger metabolic activity, one of the internal factors for the fast growth of large fish may be related to the lower expression of *IGFBP1 and IGFBP7*.

The binding protein of IGFs genes (IGFBPs) are known to inhibit cell growth and differentiation by binding specifically IGFs^38,39^, which can be influenced by many factors. Previous studies have shown that fasting increases hepatic IGFBPs levels, and IGFBPs drops back to normal after refeeding^11,40^. Whether in large-scale farming or in the natural environment, the smaller individuals have lower social status, as well as mating and feeding rights^41,42^. We could speculate that it might be more difficult for small individual loach to obtain food in paddy fields culture environment, which led to its more delayed growth and development. Furthermore, the HEGs in SG fish were enriched in the pathways related to environmental information processing such as cell adhesion molecules (CAMs), ECM–receptor interaction and Rap1 signalling pathway; cellular processes such as tight junction, focal adhesion, phagosome and adherens junction. These functional pathways consist of a complex mixture of structural and functional macromolecules and have their important roles in tissue and organ morphogenesis in maintaining cell and tissue structure and functions^43–46^. Thus, our results provide additional evidence there was a lag in the development of the small fish compared to the large fish in the paddy cultivation system.

## Conclusion

In this study, RNA-seq successfully identified that the differences in transcription levels of the loaches with differential body sizes in integrated paddy field aquaculture. Compared with the slow-growing loach, the fast-growing loach haver higher expression of metabolic genes. Furthermore, the transcription level of IGFBPs was relatively low in the fast-growing loach, which were known to inhibit cell growth and differentiation. In addition, large-scale loaches growth may be through the enlargement of muscle fibers.

## Materials and methods

### Ethics statement

All experiments were approved by the Institutional Animal Care and Use Committee (IACUC) of the College of Animal Science and Technology of Sichuan Agricultural University, Sichuan, China, under permit No. DKY-S20166409. All experimental methods were performed according to relevant guidelines and regulations and the study was carried out in compliance with the ARRIVE guidelines.

### Fish culture in paddy fields

The three paddy fields used in this experiment were performed disinfection operation to kill wild aquatic animals with quicklime before the experiment. In addition, the border of the paddy fields surrounded with a net were used to block other aquatic animals from entering the pond, such as frogs, ensured that there were only experimental fish in the paddy fields.

All experimental fish with the same genetic background came from a professional aquaculture farm of Neijiang, China (N: 104°56′27.16″, E: 29°27′32.52″). The experimental fish were cultivated in three paddy fields with an approximate area of 866.67 m^2^, when their total length was nearly 4 cm in an indoor pond. The experiment was performed from July to September, and the water quality parameters were as follows: temperature was 12–24 °C; dissolved oxygen was 5.3–6.7 mg/ml; pH was 7.5–8.1. Culture density was approximately twenty thousand fish per paddy field. The fish were fed twice a day at 09:00 and 17:00 (2 ~ 4% body weight feeding rate), and were subjected to the same daily management.

### Partition of large and small size fish

**Figure 6:**
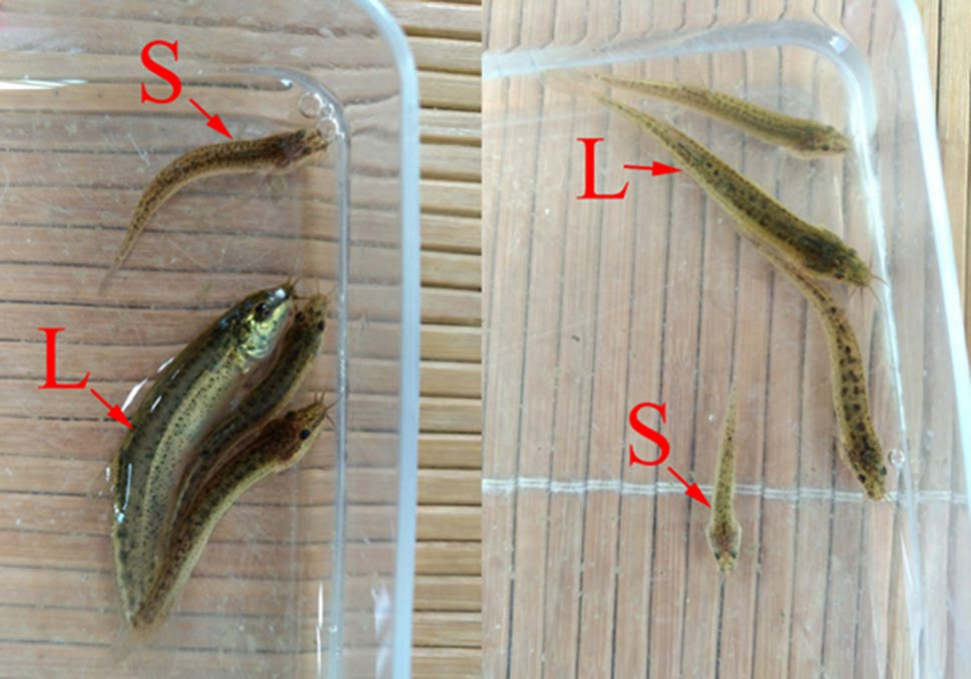
The selection of large-scale loach for LG and SG. *Note* *L* selected in LG; *S* selected in SG.

For weekly samples, the total length of fish was measured from 50 random individuals in each paddy field, and normal distributions were made based on the total length of 150 individuals. Then, top 5% of maximum total length was defined as the large group (LG); similarly, top 5% of minimum total length was defined as the small group (SG). At the twelfth week, 150 individuals were measured for the total length from each paddy field and used to divide LG and SG fish (LG and SG fish were showed in Fig. 6).

### Sample collection

Based on the definition of LG and SG fish, 3 large fish and 3 small fish were sampled weekly and fixed in fresh Bouin’s solution after being anaesthetized with MS-222 (Tricaine methane sulfonate, 100 ppm) for histological observation. For the first four weeks, the entire body of the fish was preserved from dorsal fin to caudal fin, while a piece of muscle (0.5 cm × 0.5 cm × 0.5 cm) was obtained from fish during subsequent weeks.

At the twelfth week, 3 large fish and 3 small fish from each paddy field were rapidly sampled brain (B), liver (L) and muscle (M) for RNA-Seq, after being anaesthetized using MS-222 (100 ppm). The samples were immediately frozen in liquid nitrogen, and then stored in -80 °C freezer for subsequent analysis.

### Histological characteristics and observation

Fixed samples were wrapped in gauze and dehydrated in an ethanol series, then infiltrated in xylene, and embedded in paraffin wax finally. Tissues each were cut into 6 μm sections serially using a rotary microtome according to routine procedures. Muscle sections were stained with haematoxylin and eosin (HE) for histological analysis and examined on microscope slides. Digital images were captured using a Nikon Eclipse Ti-S (Nikon Instruments Inc, Japan) and measured with Image Pro Plus software (Media Cybernetics, USA). The muscle fiber density was the number of muscle fibers per square millimetre. In detail, the muscle fibers numbers were counted within a 100 μm × 100 μm square under microscope after HE staining (200 × digital images). Only muscle fibers more than half its size within the square were counted. The muscle fiber diameter was the geometric mean of the long and short diameter of the 100 muscle fibers per fish (400 × digital images; geometric mean formula: G = $\sqrt{ab}$; G: geometric mean; a: long diameter; b: short diameter).

### Total RNA extraction and cDNA library construction

After three large fish or three small fish from each paddy filed were pooled into one sequencing sample, three LG samples and three SG samples were used sequencing, respectively. Three tissues (B: brain, L: liver and M: muscle) were used to sequence in each sample groups. Total RNA was extracted using the total RNA extraction reagent RNAiso Plus (Takara Bio Inc, Japan) according to the manufacturer’s protocol. After confirming the quality of RNA with agarose gel electrophoresis and Nanodrop 2000 (Thermo Scientific, USA), RNA that integrity number (RIN) greater than 8 and OD260/280 > 1.80 was used for mRNA library construction^47^. The qualified RNA was sent to the Annoroad Gene Technology Corporation (Beijing, China) for library preparation and sequenced using Illumina HiSeq 2000 by sequencing strategy: paired-end sequencing and raw reads length 150 bp.

### Sequencing data analysis

Low-quality, adaptor-polluted and high content of unknown bases (N)-containing sequencing reads were filtered (the adaptor sequences; unknown bases more than 10%; the percentage of no more than Q 5 bases is over 50% in a read). The Q30 of the clean data was calculated, and all downstream analyses were performed using the clean, high-quality data. Trinity (http://trinityrnaseq.sourceforge.net/, version: v2.0.6)^48^ was used to perform de novo assembly with clean reads, and Tgicl (http://trinityrnaseq.sourceforge.net/, version: v2.0.6)^49^ then used to cluster transcripts to unigenes.

After assembly, unigenes were used for functional annotation against the NT (Non-redundant protein sequences Database), NR (Nucleotide Sequence Database), Uniprot (Universal Protein), COG (Cluster of Orthologous Groups of proteins), GO (Gene Ontology) and KEGG (Kyoto Encyclopedia of Genes and Genomes) databases. Differential expression analysis was performed using edgeR^50^ in the OmicShare tools, an online platform for data analysis (www.omicshare.com/tools). The default parameters of edgeR were used, and differential expression genes (DEGs) were selected according to log_2_ (fold change) ≥ 1 and p value < 0.05. The GO and KEGG pathway analyses were then carried out with the differentially expressed genes of all the three tissues^50^.

### Validation of RNA-seq analysis by real-time PCR

Four related genes were randomly selected for quantitative real-time PCR (qRT—PCR) to identify the accuracy of RNA-seq results. 3 fish (three tissues: brain, liver, and muscle) were randomly selected from large and small group fish, respectively. *β-actin* was used as the reference gene. Total RNA was used to synthesize mRNA cDNA by using TianGen FastKing RT Kit (With gDNase). The emission intensity was detected by Step One real-time PCR system (Applied Biosystems) under the following steps: initial denaturation step at 95 °C for 20 s, 40 thermal cycling steps consisted of 3 s at 95 °C, 30 s at 60 °C. The target gene qRT-PCR primers were designed with reference to the sequences data of this study by Primer 5.0 (The reference gene primers are shown in Table S3). All reactions were run in triplicate and included no template controls for each gene and the quantitative results were quantified using the 2^−ΔΔCT^ method^51^.

### Statistical analysis

Statistical analysis was performed using SPSS 22.0 software (SPSS, Chicago, IL, USA). Data were presented as the mean ± SEM, and significant differences (*p* < 0.05) were identified using one-way analysis of variance (ANOVA)^52^.

## Supplementary Information

Supplementary Information 1.

Supplementary Information 2.

## Data Availability

Data has been deposited in the SRA under the study accession code PRJNA623189.
