# Supplementary material for: Comparative transcriptome profiles of large and small bodied large-scale loaches cultivated in paddy fields
Source: Sci Rep. 2021 Mar 2;11:4936. doi: 10.1038/s41598-021-84519-9 (PMC7925675; doi:10.1038/s41598-021-84519-9)
Supplement: Supplementary file 1 — Supplementary Information 1. [file 41598_2021_84519_MOESM1_ESM.docx]

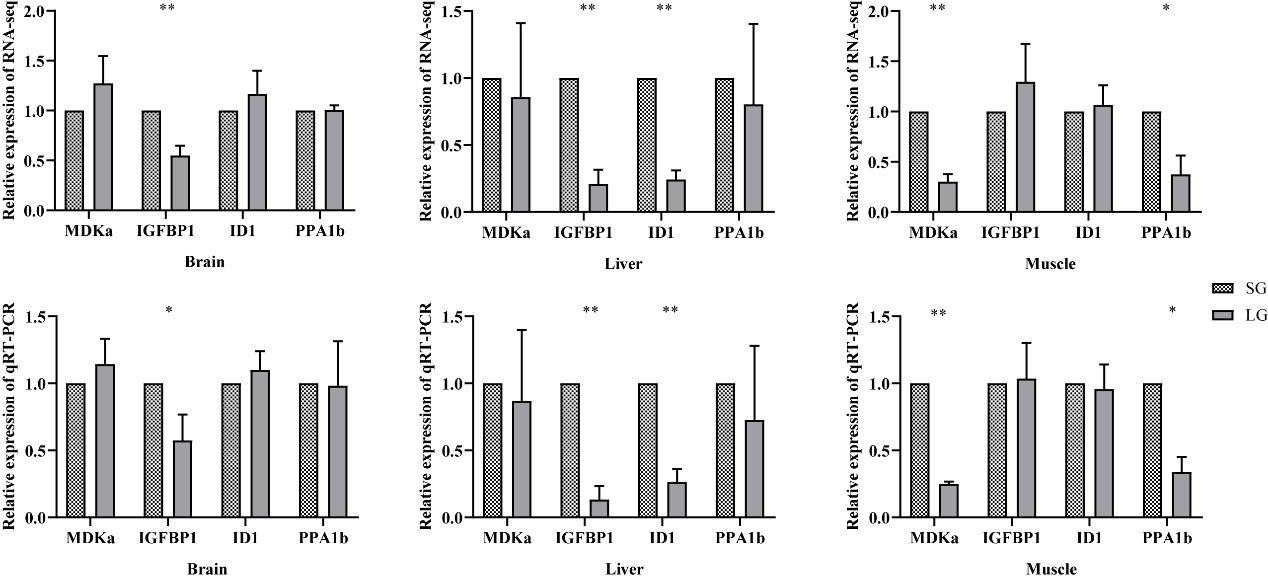


**Figure S1 Validation of DEGs with quantitative real-time PCR between LG and SG loach.** Note: Vertical bars represent mean ± SD (*n* = 6 fish individuals); * show the significant difference between LG and SG within the same gene; *: *p* < 0.05; **: *p* < 0.01.

**Table S1 The filter distribution and clean Q30 bases rate of the sequencing data.**

| Samples | Raw Reads Number (Mb) | Raw Bases Number (Gb) | Clean Reads Number (Mb) | Clean Bases Number (Gb) | Clean Reads Rate (%) | Adapter Polluted Reads Rate (%) | Ns Reads Rate (%) | Low-quality Reads Rate (%) | Raw Q30 Bases Rate (%) | Clean Q30 Bases Rate (%) |
| --- | --- | --- | --- | --- | --- | --- | --- | --- | --- | --- |
| LB1 | 53.76 | 8.06 | 44.22 | 6.63 | 82.24 | 0.94 | 0.01 | 16.81 | 89.66 | 94.4 |
| LB2 | 54.98 | 8.25 | 45.06 | 6.76 | 81.96 | 1.03 | 0.01 | 17.01 | 89.53 | 94.28 |
| LB3 | 55.93 | 8.39 | 46.68 | 7.00 | 83.45 | 1.22 | 0.01 | 15.32 | 90.26 | 94.63 |
| LL1 | 56.39 | 8.46 | 47.36 | 7.10 | 83.98 | 0.86 | 0.01 | 15.15 | 90.28 | 94.71 |
| LL2 | 53.86 | 8.08 | 45.48 | 6.82 | 84.43 | 0.81 | 0.01 | 14.75 | 90.29 | 94.53 |
| LL3 | 57.74 | 8.66 | 47.72 | 7.16 | 82.65 | 0.86 | 0.01 | 16.48 | 89.63 | 94.33 |
| LM1 | 53.08 | 7.96 | 44.73 | 6.71 | 84.26 | 1.22 | 0.01 | 14.52 | 90.56 | 94.74 |
| LM2 | 54.56 | 8.18 | 45.81 | 6.87 | 83.98 | 1.17 | 0.01 | 14.84 | 90.46 | 94.77 |
| LM3 | 55.17 | 8.27 | 46.14 | 6.92 | 83.63 | 1.07 | 0.01 | 15.29 | 90.32 | 94.74 |
| SB1 | 54.02 | 8.10 | 44.35 | 6.65 | 82.1 | 0.98 | 0.01 | 16.91 | 89.59 | 94.32 |
| SB2 | 56.66 | 8.50 | 47.31 | 7.10 | 83.5 | 0.88 | 0.01 | 15.61 | 90.08 | 94.49 |
| SB3 | 56.89 | 8.53 | 46.47 | 6.97 | 81.68 | 0.87 | 0.01 | 17.43 | 89.33 | 94.2 |
| SL1 | 54.02 | 8.10 | 43.92 | 6.59 | 81.3 | 0.74 | 0.01 | 17.95 | 89.06 | 94.09 |
| SL2 | 53.68 | 8.05 | 44.27 | 6.64 | 82.48 | 0.95 | 0.01 | 16.57 | 89.76 | 94.5 |
| SL3 | 52.84 | 7.93 | 44.82 | 6.72 | 84.82 | 0.91 | 0.01 | 14.27 | 90.74 | 94.9 |
| SM1 | 56.80 | 8.52 | 45.83 | 6.87 | 80.68 | 0.97 | 0.01 | 18.34 | 89.04 | 94.25 |
| SM2 | 57.68 | 8.65 | 47.61 | 7.14 | 82.55 | 1.23 | 0.01 | 16.21 | 89.93 | 94.59 |
| SM3 | 56.19 | 8.43 | 46.44 | 6.97 | 82.64 | 1.06 | 0.01 | 16.3 | 89.76 | 94.41 |

Note: LB, LL, LM: the brain, liver, and muscle of large group fish. SB, SL, SM: the brain, liver, and muscle of small group fish

**Table S2 Summary of the data from RNA-seq trinity assemble.**

| Length | Transcripts | |  | Unigene | |
| --- | --- | --- | --- | --- | --- |
|  | Total number | Percentage (%) |  | Total number | Percentage (%) |
| [200:400) | 92033 | 38.75 |  | 62739 | 49.38 |
| [400:600) | 36671 | 15.44 |  | 19756 | 15.55 |
| [600:1000) | 37512 | 15.79 |  | 15947 | 12.55 |
| [1000:2000) | 42996 | 18.1 |  | 15691 | 12.35 |
| [2000:--) | 28298 | 11.92 |  | 12929 | 10.17 |
| Count | 237510 |  |  | 127062 |  |
| N50 | 1600 |  |  | 1546 |  |
| N90 | 367 |  |  | 302 |  |
| Min | 201 |  |  | 201 |  |
| Max | 34240 |  |  | 34240 |  |
| Mean | 940.26 |  |  | 819.82 |  |
| Percent GC (%) | 41.5 |  |  | 40.9 |  |

**Table S3 The Primers for confirming the RNA-seq data by qRT-PCR.**

| Target genes | Sequences of primers (5′→3′) | Products length(bp) |
| --- | --- | --- |
| ID1 | F: TGAAGAGCAAAGTCGGCG | 169 |
|  | R: TTGGTCGGTATTGTGGGC |  |
| MDKa | F: GGGTGACTTGTCCAAACACTACT | 192 |
|  | R: CCTCGCTCGCTTTCTATCTATTA |  |
| IGFBP1 | F: TGCCACCCAGACAAACTTC | 95 |
|  | R: TTCTTTCACTTCAGCCCGTAT |  |
| PPA1b | F: ATCCAGTTCGTGCCGAGGTT | 184 |
|  | R: TTTCGTTGCGTCATTCATTAGC |  |
| β-actin | F: CTACGAGCTTCCTGACGGACA | 103 |
|  | R: ACCGCAAGACTCCATACCCAG |  |
